# Insight Into the Beneficial Role of *Lactiplantibacillus plantarum* Supernatant Against Bacterial Infections, Oxidative Stress, and Wound Healing in A549 Cells and BALB/c Mice

**DOI:** 10.3389/fphar.2021.728614

**Published:** 2021-11-04

**Authors:** Ashish Kumar Dubey, Mansi Podia, Priyanka, Sachin Raut, Sanpreet Singh, Anil Kumar Pinnaka, Neeraj Khatri

**Affiliations:** ^1^ IMTECH Centre for Animal Resources and Experimentation (iCARE), Council of Scientific and Industrial Research-Institute of Microbial Technology (CSIR-IMTECH), Chandigarh, India; ^2^ Academy of Scientific and Innovative Research (AcSIR), Ghaziabad, India; ^3^ Immunology Laboratory, Council of Scientific and Industrial Research-Institute of Microbial Technology (CSIR-IMTECH), Chandigarh, India; ^4^ MTCC-Microbial Type Culture Collection and Gene Bank, Council of Scientific and Industrial Research-Institute of Microbial Technology (CSIR-IMTECH), Chandigarh, India

**Keywords:** oxidative stress, wound healing, inflammation, cell-free supernatant, A549 cells, BALB/c mice, *Lactiplantibacillus plantarum*

## Abstract

*Lactiplantibacillus plantarum* MTCC 2621 is a well-characterized probiotic strain and is reported to possess many health benefits. However, the wound healing potential of this probiotic is yet to be explored. Here, we have assessed the antibacterial, antioxidant, and wound healing activities of cell-free supernatant of *Lactiplantibacillus plantarum* MTCC 2621 (Lp2621). Lp2621 exhibited excellent antibacterial activity against the indicator bacteria in the agar well diffusion assay. Lp2621 did not show any hemolytic activity. The safety of Lp2621 gel was established using the skin irritation assay in BALB/c mice, and no dermal reactions were observed. The supernatant showed 60–100% protection of A549 cells against H_2_O_2_-induced stress. In the scratch assay, Lp2621 accelerated wound healing after 24 h of treatment. The percent wound healing was significantly higher in cells treated with Lp2621 at 18–24 h posttreatment. In an excision wound healing in mice, topical application of Lp2621 gel showed faster healing than the vehicle- and betadine-treated groups. Similar wound healing activity was observed in wounds infected with *Staphylococcus aureus*. Histological examination revealed better wound healing in Lp2621-treated mice. Topical treatment of the wounds with Lp2621 gel resulted in the upregulation of pro-inflammatory cytokine IL-6 in the early phase of wound healing and enhanced IL-10 expression in the later phase. These findings unveil a protective role of Lp2621 against bacterial infection, oxidative stress, and wound healing.

## 1 Introduction

Wound healing is a multifaceted biological process involving many extracellular and intracellular macromolecules. Healing occurs in four steps: hemostasis, inflammation, proliferation, and maturation (Vaid et al., 2020), and has a vital role in skin remodeling after injury (Mousavi et al., 2020). The economic evaluation of chronic wounds suggests that care and treatment of the wound are time-consuming and cost billions of dollars every year (Nussbaum et al., 2018). Although antibiotic therapies are in place for routine care and management of the wound, they do not cover all characteristics of wound management (Nussbaum et al., 2018). Thus, researchers and the scientific community have focused their efforts on developing an alternative strategy of using probiotics that aids in the wound healing process. The World Health Organization (WHO) defines probiotics as “live microorganisms which when administered in adequate amounts confer a health benefit on the host” (FAO/WHO 2002). Probiotics lower the risk of infectious diseases, and in combination with antibiotics, combat secondary infections (King et al., 2014) as well as reduce the incidence and severity of diarrhea associated with antibiotic therapy (Hempel et al., 2012). They primarily belong to the genus *Lactobacillus* and *Bifidobacterium* (Soccol et al., 2010), and effectively modulate the immune function of the host by maintaining the balance of the intestinal microbiota (Ouwehand et al., 2016), improve the innate immunity, and moderate the functions of dendritic cells, macrophages, and T and B lymphocytes (Georgieva et al., 2015). In addition, they have also been shown to promote wound healing and modulate the inflammation caused by the pathogens through the toll-like receptor-controlled pathways (Vanderpool et al., 2008; Gholami et al., 2020). Studies have revealed that the direct application of lactic acid bacteria (LAB) on injured skin may improve skin health and augment its capacity to fight against various diseases (Nole et al., 2014; Knackstedt et al., 2020). Certain strains of the *Lactobacillus* genus play an important role in the wound healing process and protect the skin against inflammation and infections by the competitive inhibition of pathogens for adhesion sites and nutrients, modulation of the host immune response, and production of cytokines and secondary metabolites such as short-chain fatty acids as well as antimicrobial peptides (Halper et al., 2003; Peral et al., 2009; Sonal Sekhar et al., 2014; Lukic et al., 2017; Ong et al., 2020). The strain *Lactobacillus plantarum* MTCC 2621 {now renamed as *Lactiplantibacillus plantarum* [*Lpb. plantarum*] (Zheng et al., 2020)} used in this study (equivalent to *Lpb. plantarum* ATCC 8014) has been characterized for its probiotic properties (Tambekar and Bhutada 2010; Sreevani and Kumari 2013; Pop et al., 2016; Malakar et al., 2017; Khalil et al., 2018; Monteiro et al., 2019). *Lpb. plantarum* MTCC 2621 exhibited immunomodulatory activity *via* the downregulation of pro-inflammatory cytokines (Goad et al., 2013), whereas competitive inhibition with pathogens and the production of antimicrobial agents resulted in the pro-fertility property (Bhandari and Prabha 2015). The beneficial roles of this probiotic have been reported in various diseases; however, the efficacy of L*pb. plantarum* MTCC 2621 has not yet been fully elucidated in wound healing. Therefore, in this study, we assessed the antibacterial, antioxidant, and wound healing properties of the cell-free supernatant of *Lpb. plantarum* MTCC 2621 (henceforth read as Lp2621) using A549 cells *in vitro* and in a mouse model of wound healing.

## 2. Materials and Methods

### 2.1 Chemicals

De Man, Rogosa, Sharpe MRS agar, MRS broth, and nutrient agar were purchased from HiMedia, and Triton X-100; carboxymethyl cellulose, and 3-(4,5-dimethylthiazol-2-yl)-2,5-diphenyltetrazolium bromide were purchased from Sigma. The reagents for cell culture media, RPMI, and fetal bovine serum (FBS) were obtained from GIBCO. H_2_O_2_ used to induce oxidative stress was purchased from Merck.

### 2.2 Cell Line

A549 cell line (human lung carcinoma cell line) was purchased from the National Centre for Cell Science (NCCS), Pune, India.

### 2.3 Bacterial Cell Culture

*Lactiplantibacillus plantarum* MTCC 2621, *Staphylococcus aureus* MTCC 737, *Micrococcus luteus* MTCC 106, *Pseudomonas aeruginosa* MTCC 1934, *Bacillus subtilis* MTCC 441, *Escherichia coli* MTCC 739, and *Klebsiella pneumoniae* MTCC 618 were obtained from the Microbial Type Culture Collection (MTCC) (CSIR-Institute of Microbial Technology, Chandigarh, India). The culture of *Lpb. plantarum* was grown in MRS broth at 37°C with 1% (v/v) inoculum. The culture was centrifuged at 5000 rpm for 10 min, and the cell-free supernatant was collected and used in this study. All other strains were grown on the nutrient agar media at 37°C for 24 h.

**FIGURE 1:**
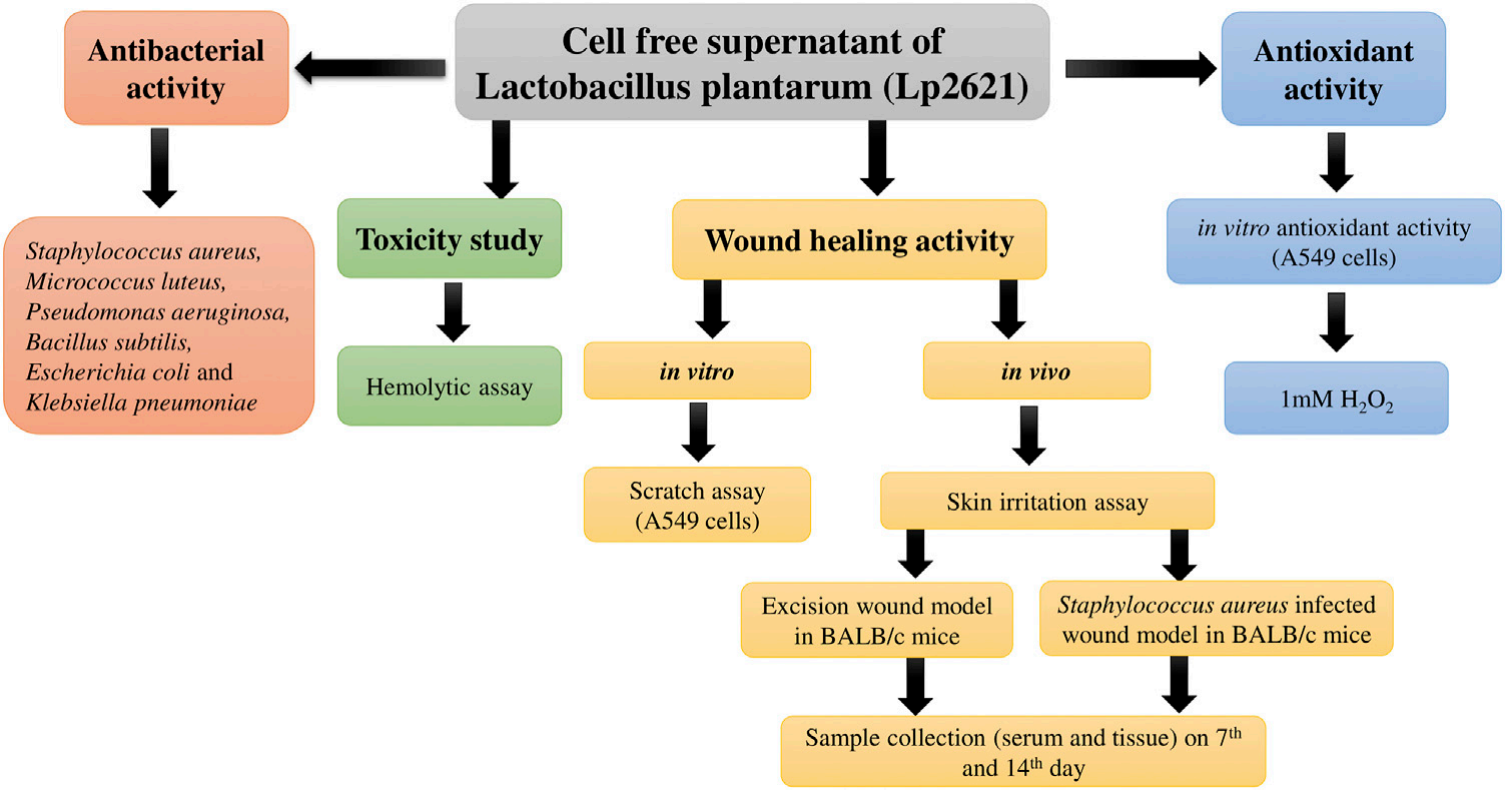
Flowchart depicting the study design for evaluation of antibacterial, antioxidant, and wound healing potential of Lp2621.

The flowchart of the experimental design of the study is illustrated in Figure 1.

### 2.4 *In Vitro* Studies

#### 2.4.1 Antibacterial Activity of Lp2621

The antibacterial property of Lp2621 was determined using the agar well diffusion assay as per previous studies (Dahiya and Purkayastha 2012). Six indicator strains were used viz. *S. aureus* (MTCC 737), *M. luteus* (MTCC 106), *P. aeruginosa* (MTCC 1934), *B. subtilis* (MTCC 441), *E. coli* (MTCC 739), and *K. pneumoniae* (MTCC 618). MRS broth alone was used as the negative control.

#### 2.4.2 Hemolytic Assay for Toxicity Testing of Lp2621

Blood collected from New Zealand white rabbit was centrifuged, washed three times with 1X PBS, and resuspended in phosphate-buffered saline (PBS) at a concentration of 4% (vol/vol). The hemolytic assay was performed as described by Jangra et al. (2019) using different concentrations of Lp2621 (0.78–100 percent in two fold dilutions). Triton X-100 (0.1%), MRS broth, and PBS were used as positive, vehicle, and negative controls, respectively.

Percent hemolytic activity was calculated using a formula.

$$\text{\% Hemolysis}=\frac{\text{Absorbance of Test}-\text{Absorbance of Blank}}{\text{Absorbance of positive control }-\text{Absorbance of Blank}}X\;100.$$ (1)

#### 2.4.3 Scratch Assay to Evaluate the Wound Healing Ability of Lp2621

The scratch assay was carried out according to Vaid et al. (2020), with slight modifications. A549 cells (1 × 10^6^ cells/well) were grown in the RPMI culture medium with 10% FBS in six-well plates and incubated overnight at 37°C in a humidified CO_2_ incubator. After incubation, the medium was removed completely, and a scratch was created on the adherent cell layer in each well by using a sterile 200 μl pipette tip. The wells were washed with 1X PBS to remove cellular debris. The RPMI medium having Lp2621 (grown up to 12 h) at a dose of 12.5 and 6.25% was added to respective wells. The positive and solvent controls received RPMI supplemented with 10% FBS and RPMI with 10% FBS and 12.5% MRS broth (used to prepare Lp2621), respectively. In negative control wells, only the RPMI medium was added. Photographs of the scratch area (wound area) were captured at 0,  6, 12, 18, and 24 h by using a trinocular microscope having a in-built camera. Data were evaluated to calculate the percent wound area using ImageJ software (LOCI, the University of Wisconsin).

#### 2.4.4 Beneficial Role of Lp2621 on H_2_O_2_-Induced Oxidative Stress in A549 Cells

Antioxidant activity of Lp2621 was evaluated by an assay in which oxidative stress was induced in A549 cells by H_2_O_2_ and the cell viability was evaluated by MTT assay according to Vaid et al. (2020), with slight modifications. Various concentrations of Lp2621 (0.78, 1.56, 3.125, 6.25, 12.5, 25, 50, and 100%) diluted in RPMI having 10% FBS and 1% pen-strep were used to treat cells in different protocols as mentioned below:

a. Concomitant exposure of A549 cells to both Lp2621 and 1.0 mM H_2_O_2_ for 24 h.
b. Exposure of A549 cells to 1.0 mM H_2_O_2_ for 4 h followed by Lp2621 treatment for 24 h.
c. 24  h pretreatment of the A549 cells with Lp2621 followed by exposure of 1.0 mM H_2_O_2_ for 4 h.

### 2.5 Preparation of Gel Containing Lp2621

*Lpb. plantarum* (MTCC 2621) was cultured in MRS broth at 37°C. The culture with 1 × 10^9^ CFU/ml was centrifuged at 5000 rpm for 10 min, and the cell-free supernatant was collected. The gel was formulated by adding 2% carboxy methyl cellulose (CMC) to the supernatant and was mixed thoroughly at room temperature until the uniform gel was formed, and stored at 4°C for further use.

### 2.6 *In Vivo* Analysis

#### 2.6.1 Animals

Eight-week-old BALB/c mice (19–25 gm weight) were taken from the IMTECH Centre for Animal Resources and Experimentation (iCARE) facility of the institute. Mice were housed in individually ventilated cages under controlled conditions of temperature (24–25°C), light (photoperiod of 12:12), and humidity (30–70%), and were provided pelleted diet and water *ad libitum*. Before starting the experiment, randomization of the animals was done, and the mice were left for a week prior to the experiment for acclimatization. The study protocol was approved by the Institutional Animal Ethics Committee (IAEC) of CSIR-Institute of Microbial Technology (IAEC approval number IAEC/20/18) and performed as per the principles and guidelines of the Committee for the Purpose of Supervision of Experiments on Animals (CPCSEA), Ministry of Fisheries, Animal Husbandry and Dairying, India.

#### 2.6.2 Skin Irritation Assay

Skin irritation assay was performed to evaluate the safety of Lp2621 gel in BALB/c mice (Draize et al., 1944). The dorsal back of the mice was shaved to remove hair, without damaging the skin surface, 24 h before the assay. The mice were divided into three groups (N = 3) according to the treatment plan, and each group was having three mice: Group I: 20% sodium lauryl sulfate (SLS) solution (positive control), Group II: CMC gel (negative control), and Group III: gel containing Lp2621 and housed individually. The gel was applied topically to the shaved skin area (approximately 1 cm^2^), and the applied sites were observed for any dermal reactions such as erythema and edema at 24, 48, and 72 h post-application. The mean erythema and edema scores were recorded based on their degree of severity caused by the application of gel as follows: no erythema/edema = 0, slight erythema/edema = 1, moderate erythema/edema = 2, and severe erythema/edema = 3.

#### 2.6.3 Wound Healing Activity of Lp2621

Having shown the antibacterial activity in the agar well diffusion assay and the wound healing activity of Lp2621 in a scratch assay using A549 cells, we next planned to conduct two experiments on the mice to check whether Lp2621 gel would be effective in wound healing and/or treating wounds infected with *S. aureus* infection as well. The mice were anesthetized using isoflurane (gas anesthesia). The hair on the dorsal side of the skin was removed, and the area was cleaned and disinfected using 70% ethanol. A full-thickness excision wound of 8 mm diameter was created in the skin of the dorsal part of mice with a sterile biopsy punch. The mice were randomized into three groups (N = 3) having nine mice in each group (*n* = 9) viz. vehicle control (CMC), positive control (betadine), and Lp2621 (test group). Each group of mice received topical application of the respective treatment twice a day for 21 days. The study continued up to 21 days, the images of the wounds were taken at days 0, 7, 14, and 21 of the study, and the wound area was calculated using ImageJ software. The percent wound contraction was calculated by the following formula.

$$\text{Percent wound contraction}=\;\frac{\text{Healed area}}{\text{Total area}}\;\text{X}\;100.$$ (2)

Three mice were euthanized at days 7 and 14 after treatment from each group. In the sham control group (*n* = 3), a wound was created without applying any treatment, and mice were euthanized after 24 h. Wound tissues were collected from different groups of mice and fixed in 10% neutral buffer formalin (NBF) solution for histopathological studies. Blood was collected from mice of different groups, and the serum was isolated and stored at −80°C for cytokine analysis.

#### 2.6.4 Efficacy of Lp2621 Gel on an Excision Wound Healing Model Infected With *S. aureus*

The same procedure for induction of a full thickness excision wound in the skin of the dorsal part of the mice was followed as mentioned under 2.6.3. Mice (*n* = 9) were divided into three groups. The grown *S. aureus* culture was centrifuged at 5,000 g for 10 min, the media was discarded, and the pellet was washed twice with PBS. Bacterial infection was initiated by placing a droplet containing 10^7^ CFU cells on the excision wound as created earlier. The treatment of the infectious wound was started 4 h postinfection. Group I was treated with CMC as the negative control, group II with betadine, and group III with Lp2621 gel. The gross images of wounds were recorded and analyzed to calculate the percent wound contraction as described above. The mice of each group (*n* = 3) were euthanized at days 7 and 14 of wounding. Blood samples and wound tissues were collected for analysis of pro- and anti-inflammatory cytokines in serum and histopathological examination, respectively.

#### 2.6.5 Histopathology

Formalin-fixed wound tissues were processed and dehydrated with graded alcohol, cleared in xylene, and molded in paraffin. Sections of 4–5 μm thickness were prepared and stained with hematoxylin and eosin (H&E), and observed under a light microscope. The H&E staining was used to evaluate fibroblast proliferation, vascularization, re-epithelization, collagen deposition, granulation of tissue formation, and the infiltration of polymorphonuclear leukocytes (PMNL).

#### 2.6.6 Cytokine Analysis

The cytokines (IL-6 and IL-10) were analyzed using a standard ELISA method. In a 96-well plate, primary antibodies, namely, IL-6 (2 μg/ml) and IL-10 (2 μg/ml), were coated in phosphate buffer (pH-9.2) and left overnight at 4°C. The next day after washing, sites were blocked with 1% bovine serum albumin for 2 h at 37°C. The plates were washed with phosphate buffer saline Tween-20, and pooled serum samples (50 µl) (dilution- 1:10) were added and incubated at 4°C overnight. Then, the biotinylated antibody was added in dilution buffer (1:1 solution of PBST and 1% BSA) and incubated at 37°C for 2 h. Streptavidin HRP (1:10,000) was added to each well, and the plate was incubated for 45 min at 37°C. Then the substrate OPD (o-phenylenediamine dihydrochloride)-H_2_O_2_ (1 mg/ml and 1 μl/ml) was added and observed for color development. The reaction was stopped using 7% H_2_SO_4_, and the reading was taken at 492 nm in an ELISA plate reader. After every step, washing was done with PBST.

### 2.7 Statistical Analysis

The results are expressed as the mean ± SE unless mentioned otherwise. All statistical analyses were done using the one-way analysis of variance (ANOVA) (SigmaPlot 11.0 program).

## 3. Results

### 3.1 Antibacterial Activity

In the agar well diffusion assay, Lp2621 exhibited distinct zones of inhibition (in mm) against all tested indicator bacterial strains viz. *S. aureus* MTCC 737 (9.03 ± 0.39), *M. luteus* MTCC 106 (18.94 ± 0.31), *P. aeruginosa* MTCC 1934 (10.53 ± 0.59), *B. subtilis* MTCC 441 (10.48 ± 0.84), and *K. pneumoniae* MTCC 618 (8.82 ± 0.39), except *E. coli*.

### 3.2 Toxicity Test Using Hemolytic Assay

**FIGURE 2:**
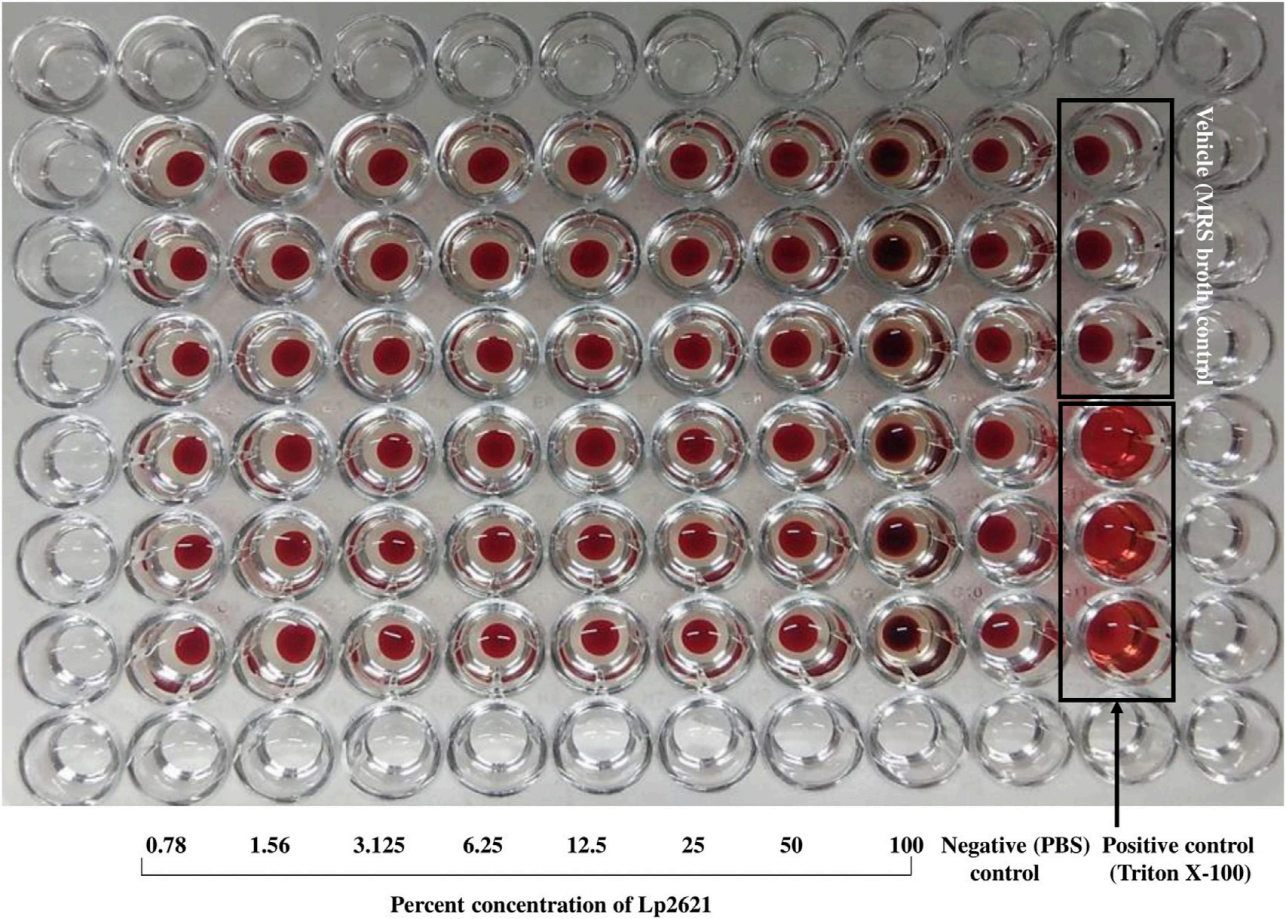
Hemolytic property of Lp2621. The image is a representative of two independent experiments performed in triplicates.

Lp2621 does not show any hemolytic activity at 0.78–100 percent concentration. In contrast, Triton X-100 (positive control) caused the complete lysis of RBCs (Figure 2).

### 3.3 Wound Healing Ability of Lp2621 in A549 Cells Using the Scratch Assay

**FIGURE 3:**
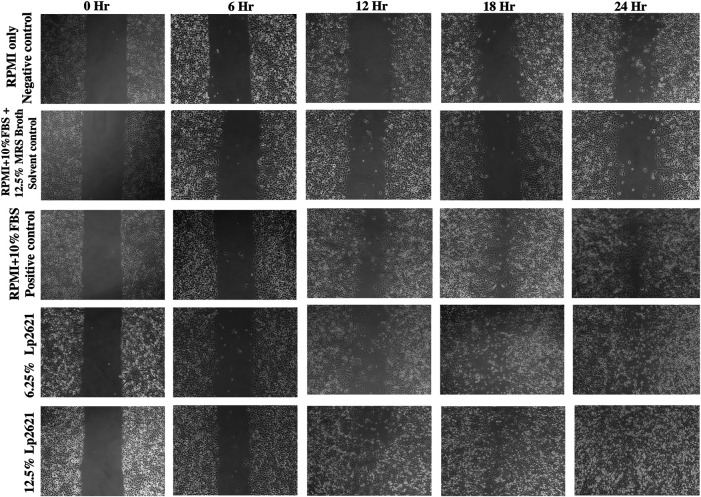
Representative microscopic images of the wound area in A549 epithelial cells in scratch assay after 0, 6, 12, 18, and 24 h incubation. Images were taken using a trinocular microscope having an in-built camera. Analysis was done using ImageJ software.

**FIGURE 4:**
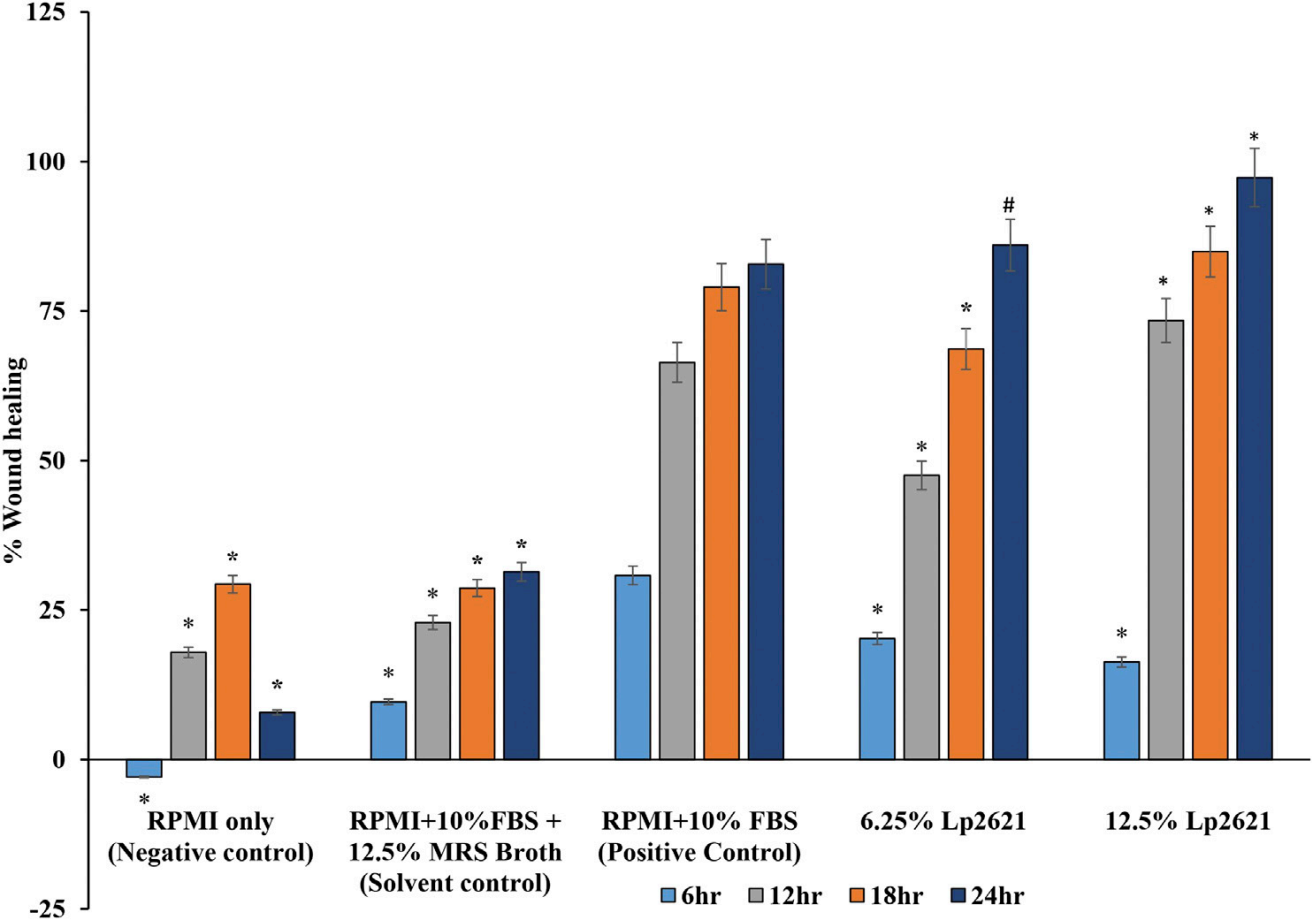
Wound healing percent (%) in scratch assay after 6, 12, 18, and 24 h post-treatment with Lp2621. Data are representative of two independent experiments performed in triplicates and expressed as mean ± SE.*, and ^#^ mean *p* < 0.001 and *p* = 0.003.

The treatment of A549 cells with different concentrations (6.25 and 12.5%) of Lp2621 results in faster wound healing than the positive control after 24 h of treatment (Figure 3). Percent wound healing is significantly higher in cells treated with Lp2621 than in various controls (*p* < 0.001 and *p* = 0.003) at 18–24 h posttreatment (Figure 4).

### 3.4 Antioxidant Activity of Lp2621

**FIGURE 5:**
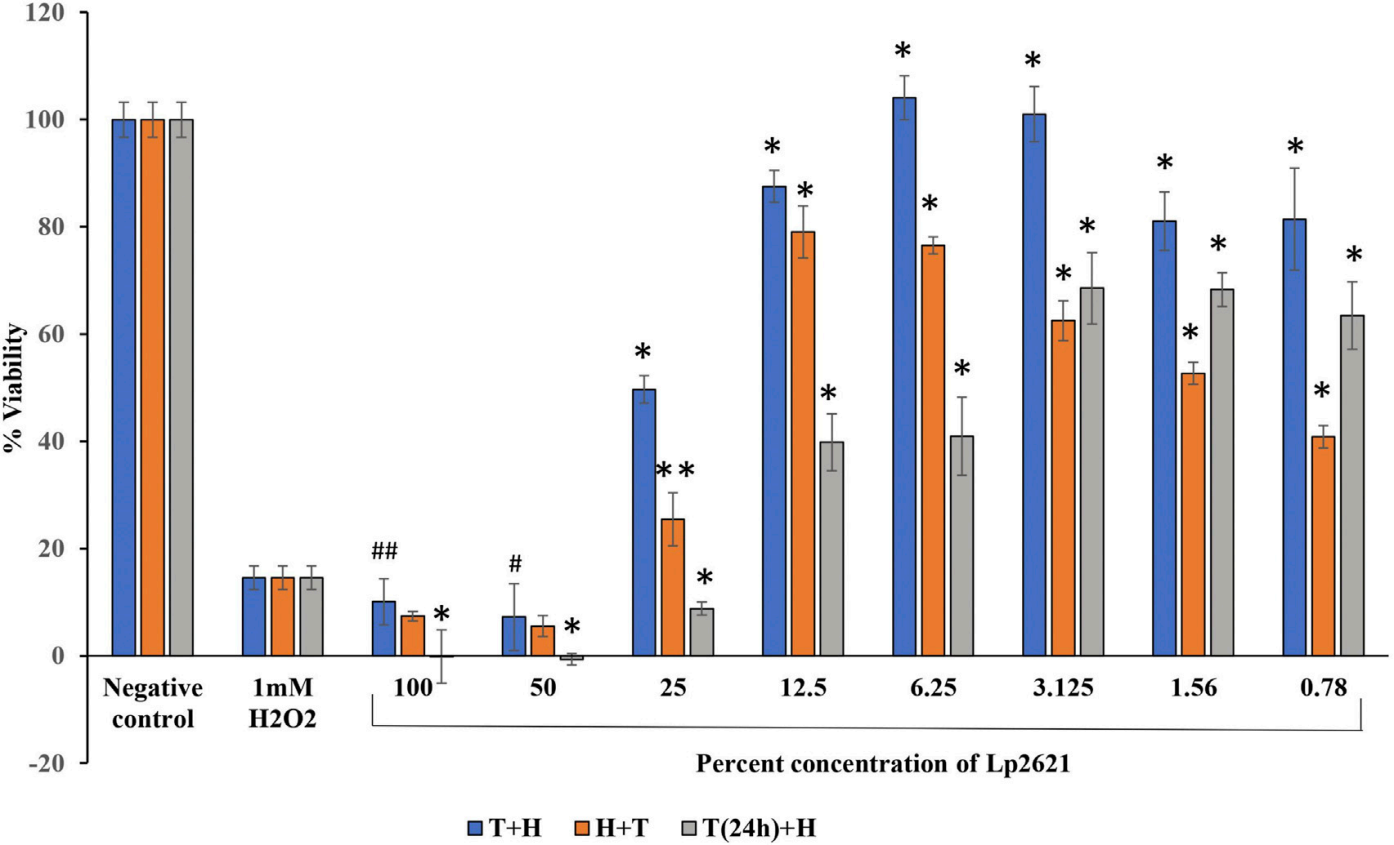
Percent viability of A549 cells treated with Lp2621.

Percent viability of A549 cells after treatment with Lp2621 and H_2_O_2_ is presented in Figure 5. Lp2621 exhibits 90–100% protection of the cells when treated at 12.5, 6.25, and 3.125% concentrations concomitantly with 1 mM H_2_O_2_ for 24 h (*p* < 0.001, *p* = 0.003 and *p* = 0.008). On the contrary, pre-exposure of cells to H_2_O_2_ for 4 h followed by treatment with Lp2621 at similar concentrations results in 60–80% cell viability (*p* < 0.001 and *p* = 0.001). Furthermore, 24 h pretreatment of cells with Lp2621 (3.125, 1.56, and 0.78%) followed by the exposure to H_2_O_2_ for 4 h results in a decline in cell viability to 60% (*p* < 0.001).

### 3.5 *In Vivo* Analysis

#### 3.5.1 Skin Irritation Assay

**FIGURE 6:**
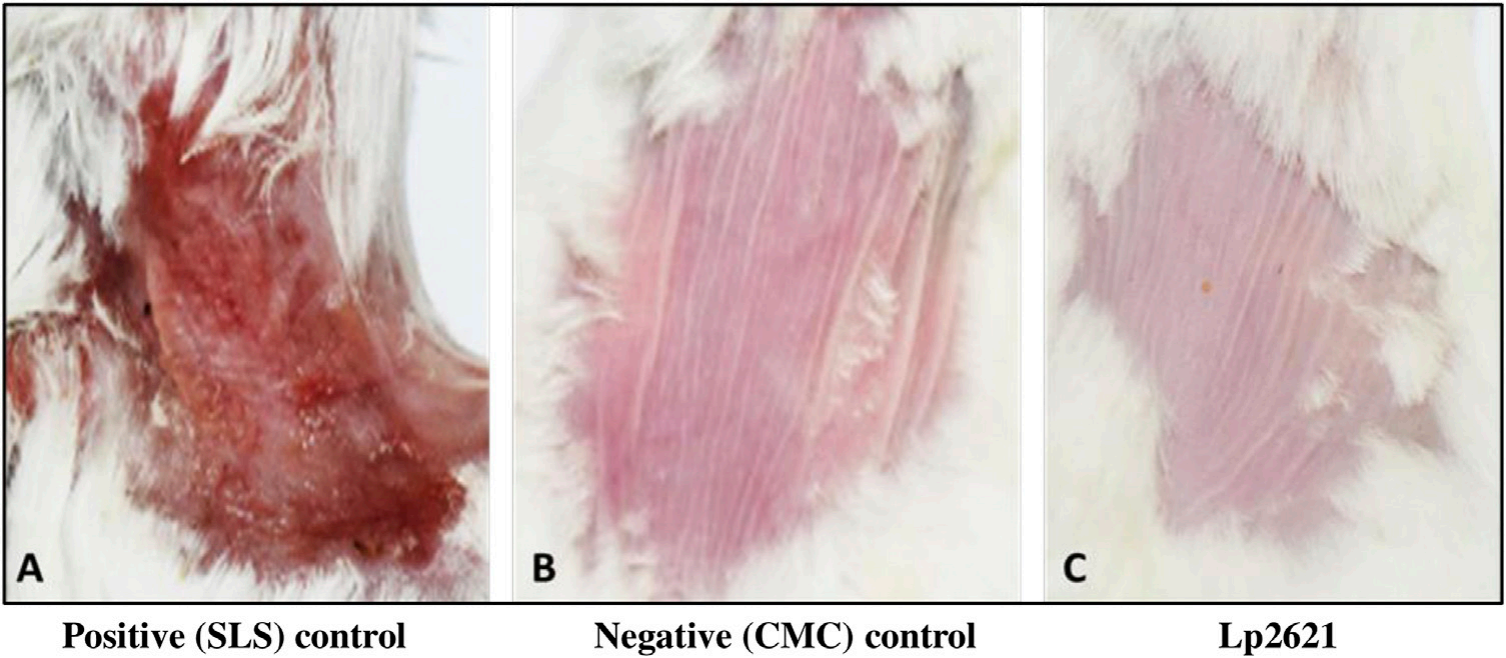
Representative images of the skin irritation assay using Lp2621 at 72 h: **(A)** positive control, SLS treated, **(B)** negative control, CMC gel, and **(C)** treated with gel containing Lp2621.

Mice treated with negative control and gel containing Lp2621 did not display any abnormal irritation even after 72 h of application, whereas mice treated with SLS exhibits severe dermal reactions such as erythema and edema at the site of application (Figure 6).

#### 3.5.2 Efficacy of Lp2621 Gel on Excision Wound Healing Model

**FIGURE 7:**
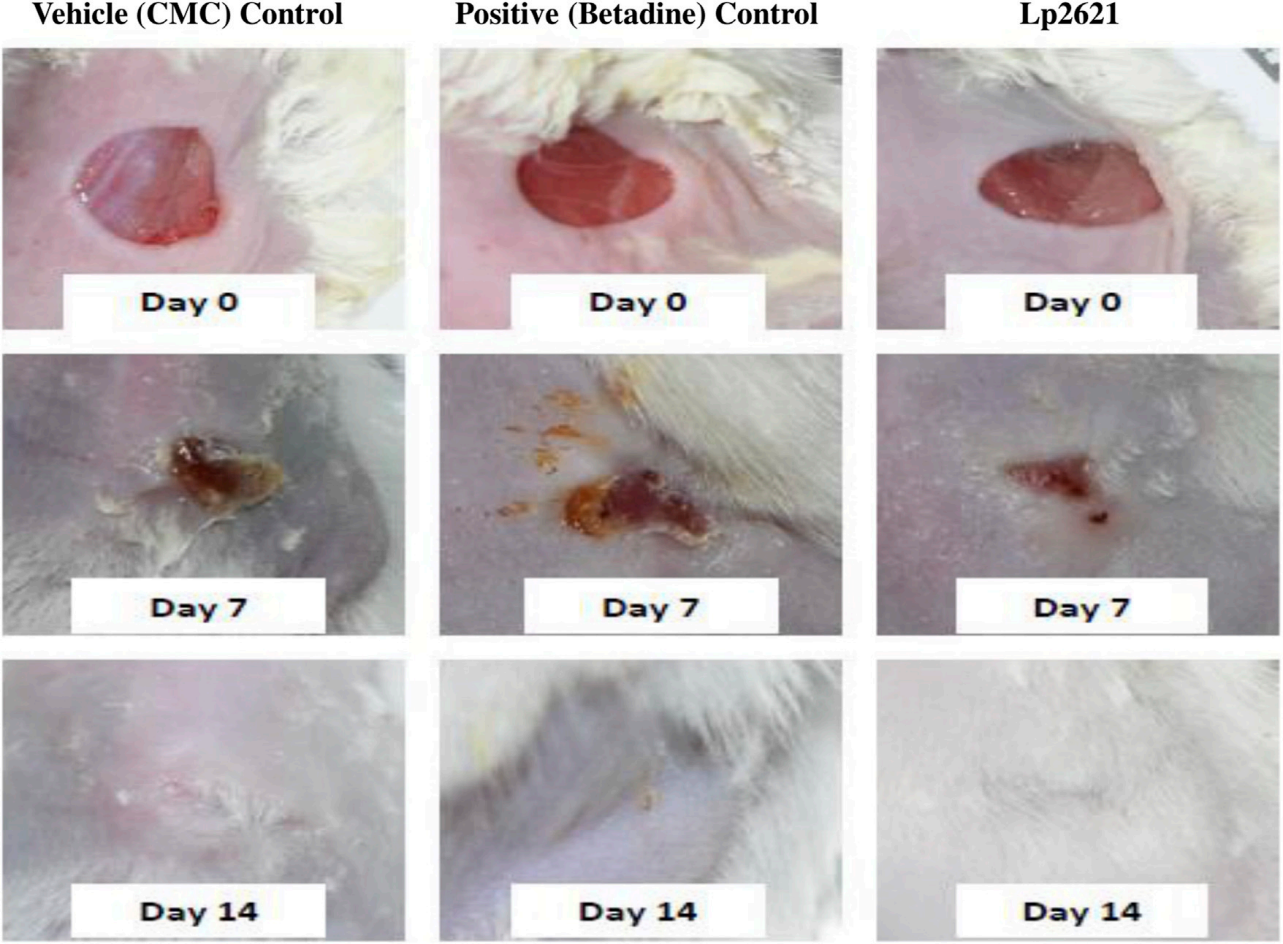
Full-thickness excision wounds were created in mice. Representative photographs from the mice showing macroscopic wound closure on different day’s post-injury.

**FIGURE 8:**
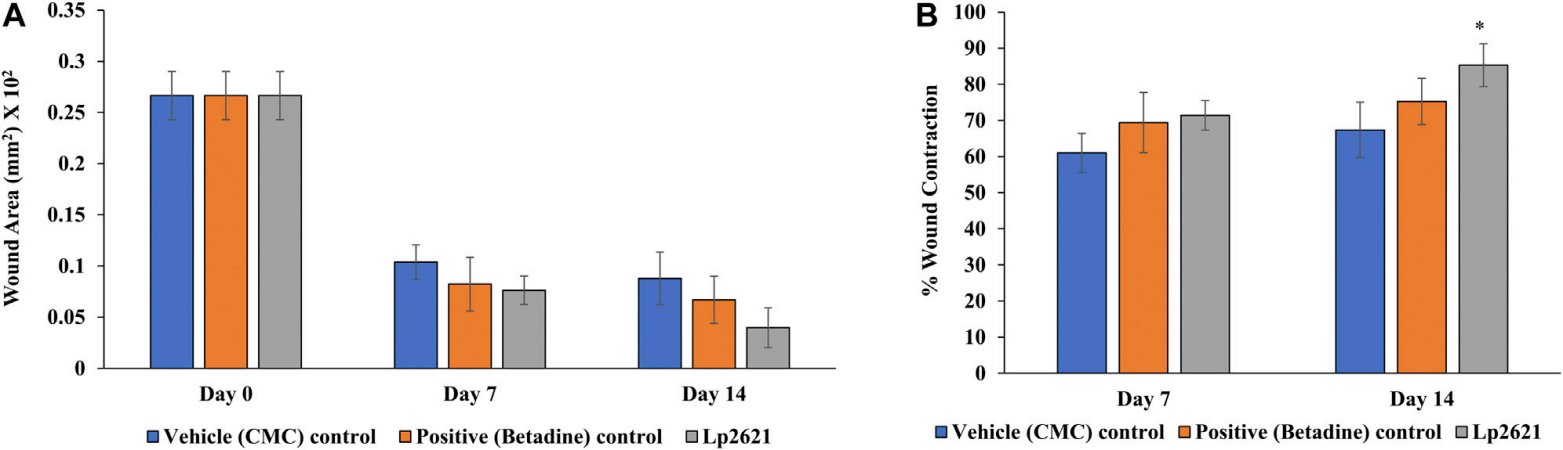
Effect of topical treatment of Lp2621 gel; **(A)** wound area and **(B)** percent wound contraction at different day’s post-wounding. Data are expressed as mean ± SD. *mean *p* = 0.033.

**FIGURE 9:**
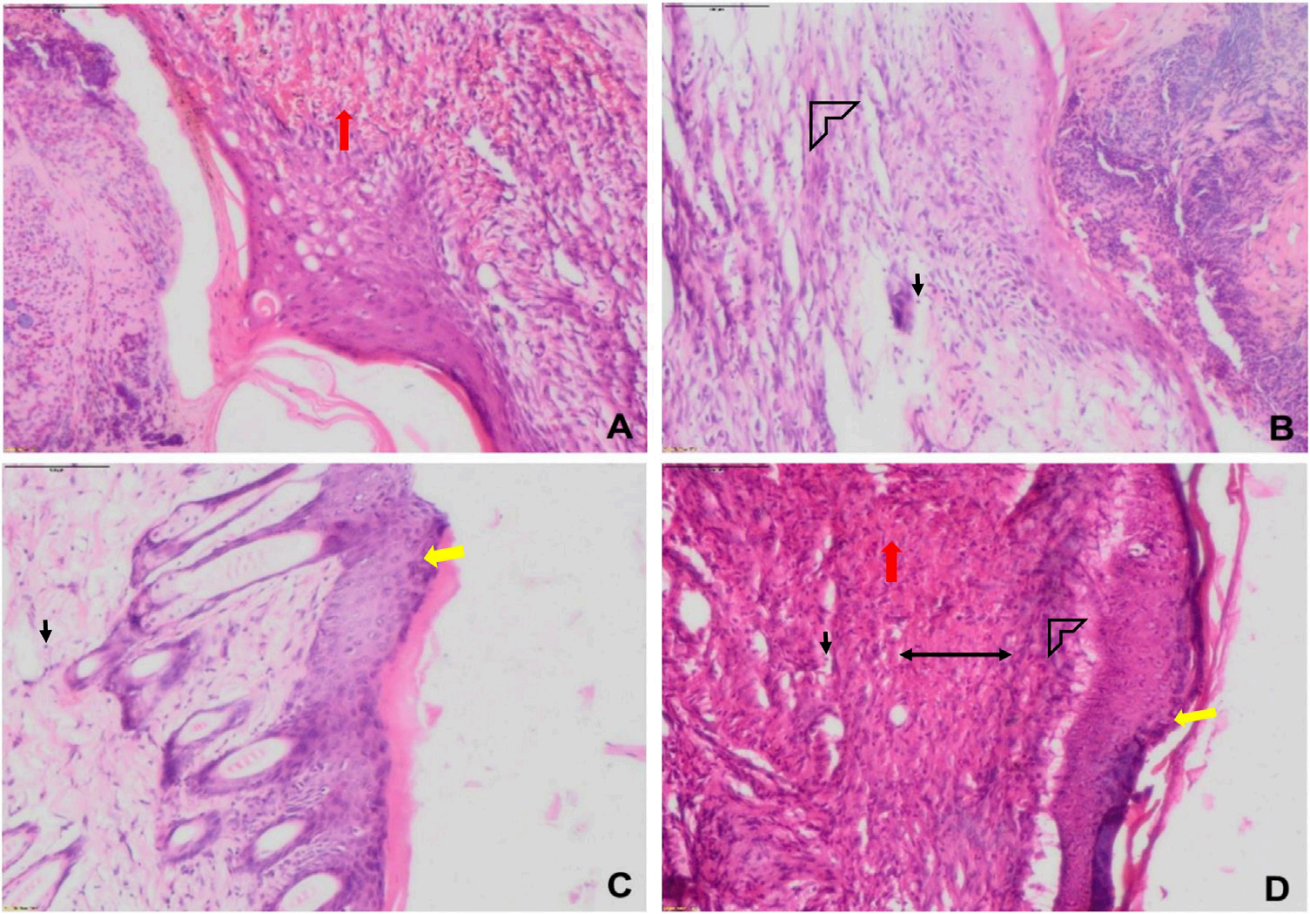
Representative histological images of wounds of various groups on day 1 **(A)** and day 7 **(B–D)**. **(A)** sham, **(B)** vehicle (CMC), **(C)** positive (betadine), **(D)** Lp2621 (scale bar = 100 μm). Fibroblasts (black), vascularization (red), re-epithelization (yellow), collagen deposition (arrow head), and granulation of tissue (double-sided arrow).

The results showed that the treatment of the wound with Lp2621 gel exhibited considerable wound healing as compared to the vehicle and betadine-treated groups of mice, and is indicated by a reduction in the wound area as well as percent contraction (*p* = 0.033) of the wound (Figure 7 and Figures 8A,B). Histopathological examination of wound tissues on day 7 (Figure 9) showed an enhanced proliferation of fibroblasts, vascularization, re-epithelization, collagen deposition, and the granulation of tissue in betadine- and Lp2621 gel–treated groups as compared to the vehicle-treated group. These results were corroborated by histopathological examination of the tissues, on day 14. The wound healing was incomplete in vehicle-treated mice, while in the betadine- and Lp2621 gel–treated groups of mice, the tissue was completely healed and appears to be histologically normal.

#### 3.5.3 Efficacy of Lp2621 Gel on an Excision Wound Healing Model Infected With *S. aureus*

**FIGURE 10:**
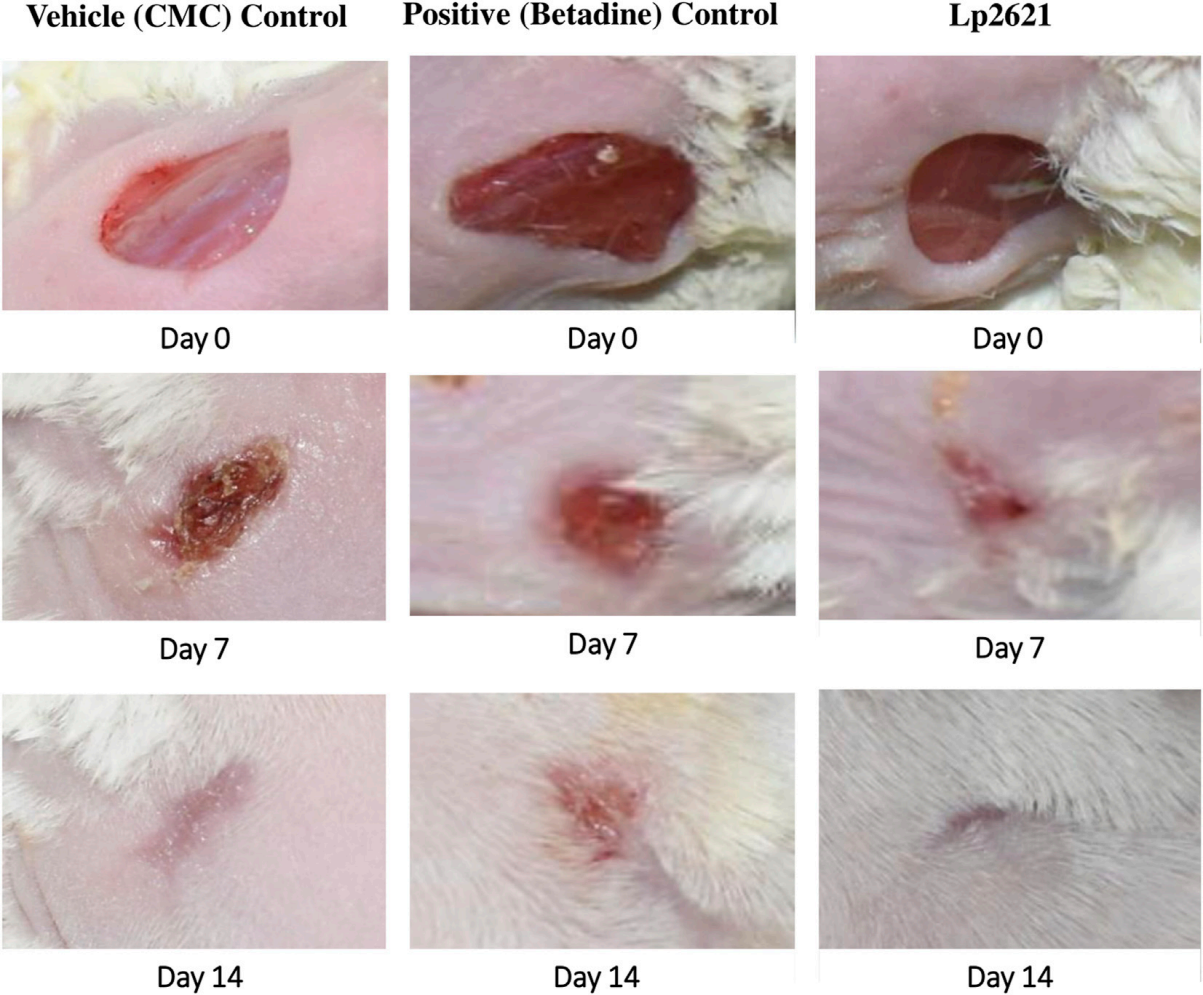
Full-thickness excision wounds were created in mice and infected with S. *aureus*. Representative photographs from mice showing macroscopic wound closure on different day’s post-injury.

**FIGURE 11:**
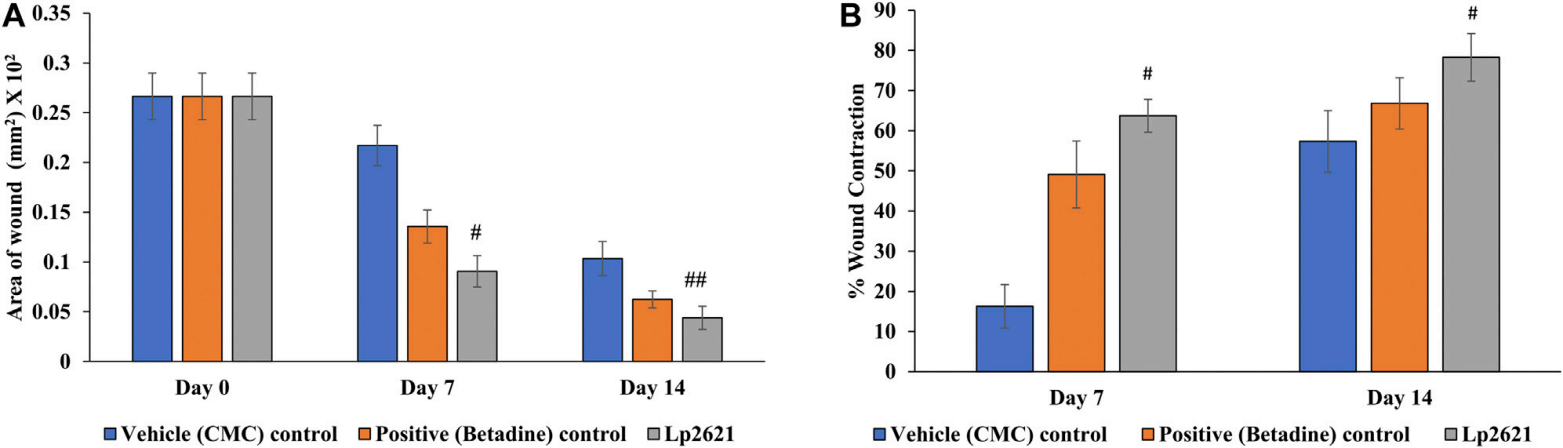
Effect of topical treatment of Lp2621 gel on different groups: **(A)** on wound area infected with S. *aureus* and **(B)** percent wound contraction at different days post-wounding. Data are expressed as mean ± SD. ^#^, and ^##^ mean *p* = 0.002, and *p* = 0.020.

**FIGURE 12:**
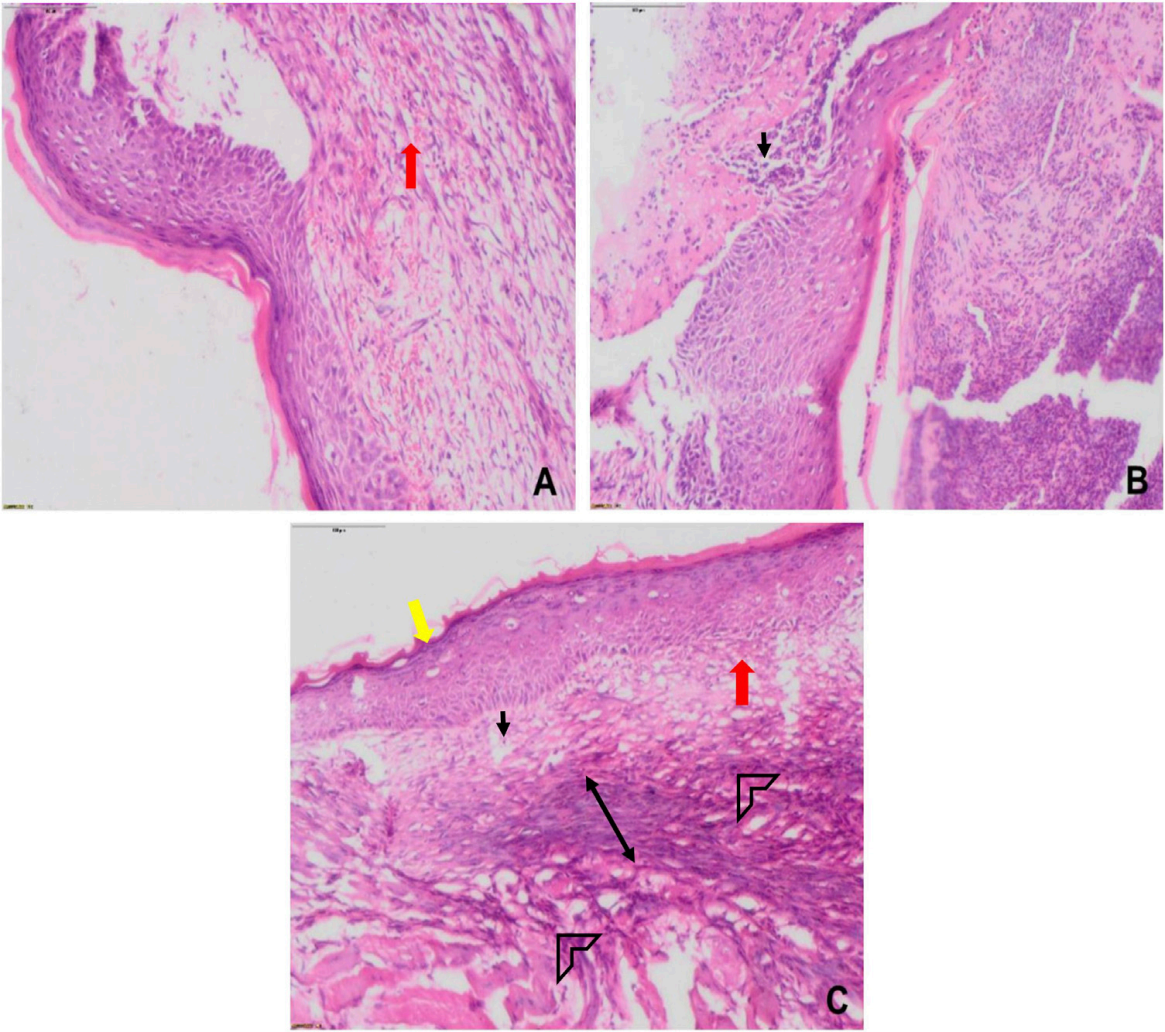
Histological images of skin tissue from infected wounds on day 7: **(A)** vehicle (CMC), **(B)** Positive (betadine), **(C)** Lp2621 (scale bar = 100 μm). Fibroblasts (black), vascularization (red), re-epithelization (yellow), collagen deposition (arrow head), and granulation of tissue (double-sided arrow).

The efficacy of Lp2621 gel on the excision wound in the mice infected with *S. aureus* was evaluated. The results show that the treatment of infected wound with Lp2621 gel leads to a substantially quicker wound recovery compared to the vehicle and positive control as evident by a reduction in the wound area and an increase in the percent contraction (*p* = 0.002 and *p* = 0.020) of the wound (Figure 10 and Figures 11A,B). The rate of wound healing activity was better in betadine- and Lp2621 gel–treated infected wounds than in the untreated infected wounds as observed on day 7 (Figure 12). The vehicle-treated infected wound tissues depict persistent inflammatory changes with the infiltration of inflammatory cells, mainly neutrophils, granulation of connective tissue in the wound area with numerous loops of blood vessels, fibroblast proliferation, and poor re-epithelization (Figure 12A). However, betadine- and Lp2621 gel–treated infected wound tissues depict re-epithelization of tissues with a reduced infiltration of leukocytes, increased fibroblastic activity, collagenation, and granulation of tissues (Figures 12B,C).

#### 3.5.4 Cytokine Analysis

**FIGURE 13:**
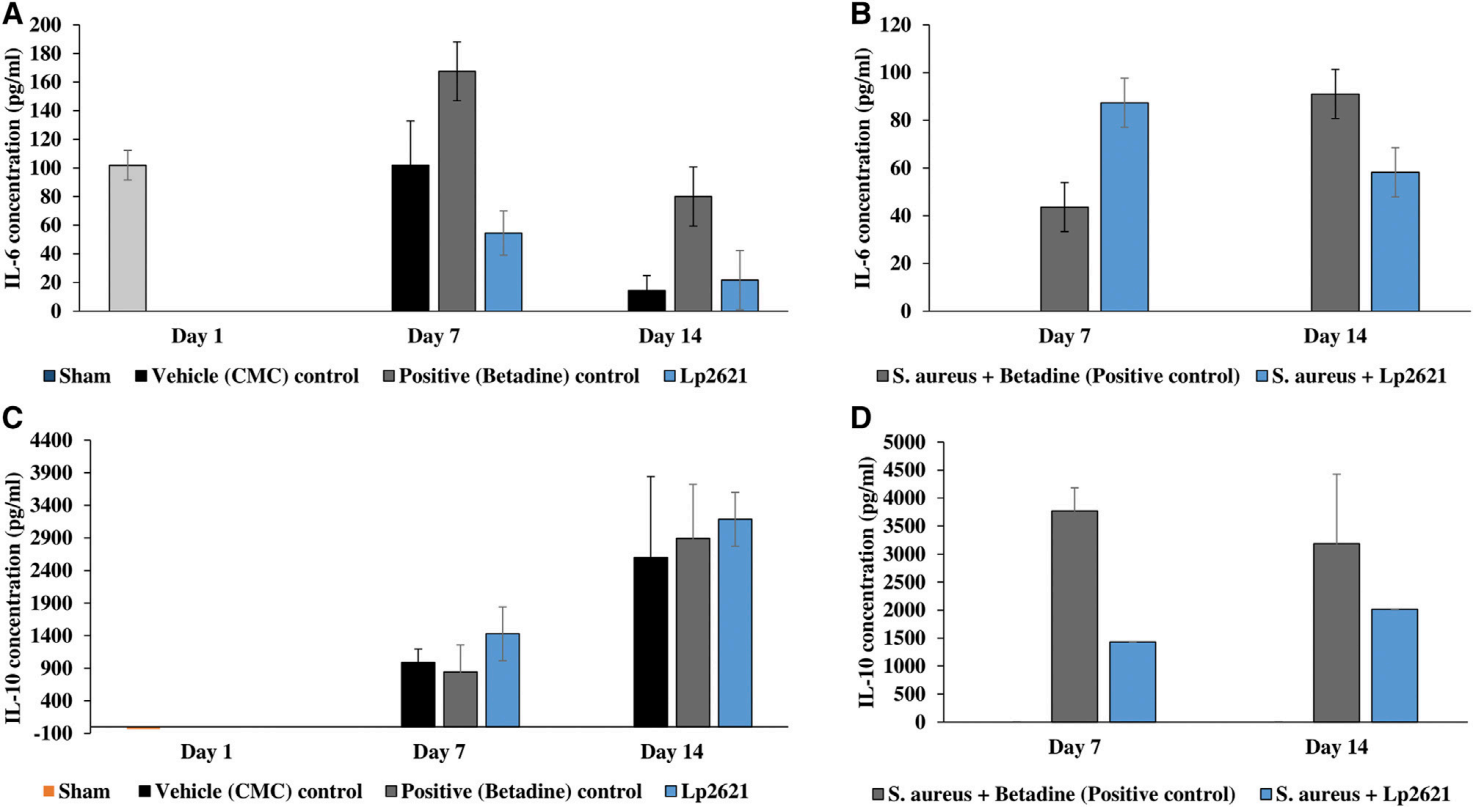
Cytokine levels in the serum sample of mice. **(A,B)** IL-6 levels in the excision wound and the wound infected with *S. aureus*. **(C,D)** IL-10 levels in the excision wound and the wound infected with *S. aureus*. Data are representative of two independent experiments performed in duplicates and expressed as mean ± SE.

We further verified the role of Lp2621 in the immunoregulation of pro- and anti-inflammatory cytokines in the healing of normal and/or wounds infected with *S. aureus* infection. As shown in Figures 13A–D, the serum levels of pro-inflammatory cytokine IL-6 were elevated in the initial phase of wound healing but declined on day 14. On the contrary, higher levels of IL-10 were observed during the later phase of wound recovery. However, this variation in levels of cytokines (IL-6 and IL-10) in normal and/or wounds infected with *S. aureus* infection was not statistically significant between the experimental groups on the respective day of the study.

## 4. Discussion

In the present study, we assessed the antibacterial, hemolytic, antioxidant, and the wound healing properties of Lp2621 in A549 cells and excision wounds with and without *S. aureus* infection model in mice. Previous studies have reported the probiotic potential (Huang et al., 2013; Pop et al., 2016; Khalil et al., 2018; Monteiro et al., 2019) and antimicrobial activity of various *Lactobacillus* species (Halder et al., 2017; Prabhurajeshwar and Chandrakanth 2019; Qian et al., 2020) as well as *Lpb. plantarum* MTCC 2621 (Sreevani and Kumari 2013). The mechanisms behind the antibacterial activity of *Lactobacillus* strains are likely due to the production of antimicrobial compounds, resistance and competition for nutrients with other pathogenic bacteria, reduction of bacterial attachment to the mucosa, and modulation of the host immune system (Maria Tufail 2011; Giani et al., 2019).

The cutaneous wound healing activity of various *Lactobacillus* species has been previously reported in animal studies (Tsiouris and Tsiouri 2017). Another group demonstrated that the topical application of live *L. reuteri* DSM 17938 and its lysate induced anti-inflammatory activity by reducing the levels of pro-inflammatory cytokines (IL-6 and IL-8) (Khmaladze et al., 2019). The antimicrobial and *in vivo* wound healing potential of the probiotic VITSAMJ1 in rats has been studied previously (Sinha et al., 2019). Similar outcomes were observed in the burn wounds, where topical application of *Lpb. plantarum* could promote the wound healing (Satish et al., 2017). Probiotics such as *Lacticaseibacillus paracasei* and *Lpb. plantarum* significantly enhanced the production of IL-6 in the presence of IL-1*β*, an inflammatory cytokine in enterocytes (Caco2 cells), intermediated through hsp70 and hsp27 heat shock proteins (Reilly et al., 2007). Our findings (Figures 7–12) are consistent with the recent work by Khodaii and coworkers, where the wound healing activity was considerably promoted by the administration of *L. reuteri* extract by day 15 post-wounding (Khodaii et al., 2019). *L. reuteri* promoted wound healing *via* the PI3K/AKT/*β*-catenin/TGF*β*1 pathway (Han et al., 2019). In another study, *Limosilactobacillus fermentum* enhanced the wound healing by promoting the production of anti-inflammatory and anti-pathogenic factors (Brandi et al., 2020). Ashoori et al. (2020) observed that the rate of wound healing was faster in the groups treated with both *L. reuteri* and *L. fermentum* supernatant-loaded chitosan nanogel (Ashoori et al., 2020). The metabolites of probiotics increased proteoglycan deposition, angiogenesis, reduced inflammation, and stimulated different growth factors (Matsumoto et al., 2005; Sonal Sekhar et al., 2014).

The histopathological examination of wound tissues in the present study (Figure 9 and Figure 12) revealed angiogenesis and the recruitment of PMNL at the site of injury. These results are consistent with the earlier findings where a subcutaneous injection of LS into the mouse caused a continuous influx of polymorphonuclear leukocytes (PMNL) and macrophages in the wound area, and stimulated the inflammatory phase of the tissue repair (Halper et al., 2003). Histological changes were characterized by the infiltration of polynuclear neutrophils and dilatation of blood vessels along with a significant decrease in serum levels of pro-inflammatory cytokines such as IL-1*β*, IL-6, TNF-α, IL-17, and IL-22, while an increase in the levels of IL-10 was observed in *Ligilactobacillus salivarius* LA307–treated mice (Holowacz et al., 2018). *Lactobacillus bulgaricus* and *Lpb. plantarum* accelerated wound healing by decreasing IL1*β* and TNF*α*, and upregulating IL-10 expression in diabetic Wistar rats (Mohtashami et al., 2020). The probiotic strains have been consistently reported to modulate the pro-inflammatory cytokine, IL-6, and upregulate the level of anti-inflammatory cytokine, IL-10 (Karamese et al., 2016; Holowacz et al., 2018; Johnson et al., 2020). The favorable histological changes observed upon the treatment of the wound area with probiotic strains/extracts such as infiltration of polynuclear neutrophils and dilation of blood vessels are concomitant to the dynamic levels observed of IL-6 and is in accordance with the reported modulatory role (Holowacz et al., 2018; Johnson et al., 2020). Also, increase in angiogenesis, tissue regeneration, matrix remodeling, and repair are corroborated to increase in IL-10 and as such help in the regenerative process (Steen et al., 2020).

The findings of our wound healing study provide evidence that the topical application of Lp2621 to infected and uninfected wounds demonstrated rapid healing *via* enhanced angiogenesis, proliferation of fibroblasts, re-epithelization, and recruitment of PMNL. Another key finding of our study is that IL-6 level was elevated in the initial phase of wound healing followed by a decline by day 14. On the contrary, higher level of IL-10 was observed during the later phase of wound healing. The findings thus underscore the importance of cell-free supernatant of probiotic bacteria, *Lpb. plantarum* 2621 in treating both normal and *S. aureus*–infected wounds. These findings, therefore, suggest that probiotics and/or their metabolites have potential for the treatment of drug-resistant bacteria. Future research will be directed toward the development of probiotics/consortia of probiotics and their metabolites as alternatives to antibiotics for the effective treatment of drug-resistant bacteria, thereby thwarting the serious global threat of antimicrobial resistance.

## Data Availability

The original contributions presented in the study are included in the article/Supplementary Material; further inquiries can be directed to the corresponding author.
